# Doubts and concerns about COVID-19 uncertainties on imaging data, clinical score, and outcomes

**DOI:** 10.1186/s12890-023-02763-3

**Published:** 2023-11-25

**Authors:** Cosimo Nardi, Andrea Magnini, Linda Calistri, Edoardo Cavigli, Anna Julie Peired, Vieri Rastrelli, Edoardo Carlesi, Giulia Zantonelli, Olga Smorchkova, Lorenzo Cinci, Martina Orlandi, Nicholas Landini, Edoardo Berillo, Chiara Lorini, Jessica Mencarini, Maria Grazia Colao, Leonardo Gori, Valentina Luzzi, Chiara Lazzeri, Elisa Cipriani, Manuela Bonizzoli, Filippo Pieralli, Carlo Nozzoli, Alessandro Morettini, Federico Lavorini, Alessandro Bartoloni, Gian Maria Rossolini, Marco Matucci-Cerinic, Sara Tomassetti, Stefano Colagrande

**Affiliations:** 1 Department of Experimental and Clinical Biomedical Sciences, Radiodiagnostic Unit n. 2, University of Florence-Careggi University Hospital, Largo Brambilla 3, 50134 Florence, Italy; 2 Department of Radiology, Careggi University Hospital, Largo Brambilla 3, 50134 Florence, Italy; 3 Department of Experimental and Clinical Biomedical Sciences “Mario Serio”, University of Florence, Largo Brambilla 3, 50134 Florence, Italy; 4 Neuroradiology Unit, Department of Radiology, Careggi University Hospital, Largo Brambilla 3, 50134 Florence, Italy; 5 Department of Experimental and Clinical Medicine, Division of Rheumatology, Careggi University Hospital, University of Florence, Largo Brambilla 3, 50134 Florence, Italy; 6 Department of Radiological, Oncological and Pathological Sciences, Policlinico Umberto I Hospital, “Sapienza” Rome University, Rome, Italy; 7 Department of Clinical and Experimental Medicine, Interventional Pulmonology Unit, Careggi University Hospital, Largo Brambilla 3, 50134 Florence, Italy; 8 Department of Health Sciences, University of Florence, Largo Brambilla 3, 50134 Florence, Italy; 9 Department of Experimental and Clinical Medicine, Infectious and Tropical Diseases Unit, Careggi University Hospital, University of Florence, Largo Brambilla 3, 50134 Florence, Italy; 10 Department of Experimental and Clinical Medicine, University of Florence, Largo Brambilla 3, 50134 Florence, Italy; 11 Clinical Microbiology and Virology Unit, Florence Careggi University Hospital, Largo Brambilla 3, 50134 Florence, Italy; 12 Intensive Care Unit and Regional ECMO Referral Centre, Azienda Ospedaliero-Universitaria Careggi, Largo Brambilla 3, 50134 Florence, Italy; 13 Intermediate Care Unit, University Hospital Careggi, Largo Brambilla 3, 50134 Florence, Italy; 14 Internal Medicine Unit 1, Careggi University Hospital, Largo Brambilla 3, 50134 Florence, Italy; 15 Internal Medicine Unit 2, Careggi University Hospital, Largo Brambilla 3, 50134 Florence, Italy; 16 Department of Experimental and Clinical Medicine, Division of Pulmonology, Careggi University Hospital, University of Florence, Largo Brambilla 3, 50134 Florence, Italy

**Keywords:** COVID-19, SARS-CoV-2, Acute respiratory disease, Pneumonia, Computed tomography

## Abstract

**Background:**

COVID-19 is a pandemic disease affecting predominantly the respiratory apparatus with clinical manifestations ranging from asymptomatic to respiratory failure. Chest CT is a crucial tool in diagnosing and evaluating the severity of pulmonary involvement through dedicated scoring systems. Nonetheless, many questions regarding the relationship of radiologic and clinical features of the disease have emerged in multidisciplinary meetings. The aim of this retrospective study was to explore such relationship throughout an innovative and alternative approach.

**Materials and methods:**

This study included 550 patients (range 25–98 years; 354 males, mean age 66.1; 196 females, mean age 70.9) hospitalized for COVID-19 with available radiological and clinical data between 1 March 2021 and 30 April 2022. Radiological data included CO-RADS, chest CT score, dominant pattern, and typical/atypical findings detected on CT examinations. Clinical data included clinical score and outcome. The relationship between such features was investigated through the development of the main four frequently asked questions summarizing the many issues arisen in multidisciplinary meetings, as follows 1) CO-RADS, chest CT score, clinical score, and outcomes; 2) the involvement of a specific lung lobe and outcomes; 3) dominant pattern/distribution and severity score for the same chest CT score; 4) additional factors and outcomes.

**Results:**

1) If CT was suggestive for COVID, a strong correlation between CT/clinical score and prognosis was found; 2) Middle lobe CT involvement was an unfavorable prognostic criterion; 3) If CT score < 50%, the pattern was not influential, whereas if CT score > 50%, crazy paving as dominant pattern leaded to a 15% increased death rate, stacked up against other patterns, thus almost doubling it; 4) Additional factors usually did not matter, but lymph-nodes and pleural effusion worsened prognosis.

**Conclusions:**

This study outlined those radiological features of COVID-19 most relevant towards disease severity and outcome with an innovative approach.

**Supplementary Information:**

The online version contains supplementary material available at 10.1186/s12890-023-02763-3.

## Introduction

Since the beginning of the SARS-CoV-2 pandemic and its disease (COVID-19), over 700 million cases of infection and 6.9 million deaths have been reported world-wide (https://covid19.who.int/?mapFilter=cases) [1]. Clinical manifestations of the infection range from asymptomatic/pauci-symptomatic disease to severe pneumonia with acute respiratory failure requiring mechanical ventilation, septic shock, and multiple organ failure [2]. Overall, 22% of infected patients will develop severe disease, whereas 11 and 7% will require admission to the Intensive Care Unit (ICU) and mechanical ventilation respectively; mortality rate is 5.6% [3]. Since SARS-CoV-2 targets predominantly respiratory and vascular systems, the Fleischner Society recommends using CT examinations in case of worsening of symptoms or environments resource-constrained for Nucleic Acid Amplification Tests (NAAT) [4]. This last point was actually true in the first months of the pandemic, when the rate of infected people exceeded the number of available tests, but the development and the spread of antigenic tests allowed to overcome this limitation [5]. To help in the diagnosis of COVID-19 disease basing on chest CT scan, two main scoring systems have been developed, namely the COVID-19 Reporting and Data System (CO-RADS) and Radiological Society of North America classification system for reporting COVID-19 pneumonia [6]. These systems showed almost overlapping values for sensitivity and specificity without being capable to predict disease severity [7]. Further scoring systems have been developed, all of them showing good correlation with disease severity [8–12]. Such systems considered the two lungs as a whole by summing scores for segments-lobes, neither exploring whether a relationship between the involvement of selective lobes and symptoms existed. Moreover, only few studies with relatively small samples [13–15] investigated the relationship between chest CT findings and COVID-19 disease severity/outcomes. Interestingly, Cereser et al. suggested that CT alone could potentially identify patients at risk of disease progression beyond currently known risk factors, after finding no relationships between comorbidities and disease severity in their series [15]. Therefore, current literature has left many questions unanswered, and many doubts have ceaselessly arisen about patients’ management and prognosis during multidisciplinary team hospital meetings (MDT) held in our Institution.

This retrospective study aimed at investigating relationships between CO-RADS, severity scoring systems, common chest CT patterns, clinical illness severity, and outcome (discharge after hospital admission in ordinary ward; discharge after admission in ICU; death) trying to answer the most frequently asked questions (FAQ) arising during MDT concerning COVID-19.

## Materials and methods

### Patients

**Fig. 1:**
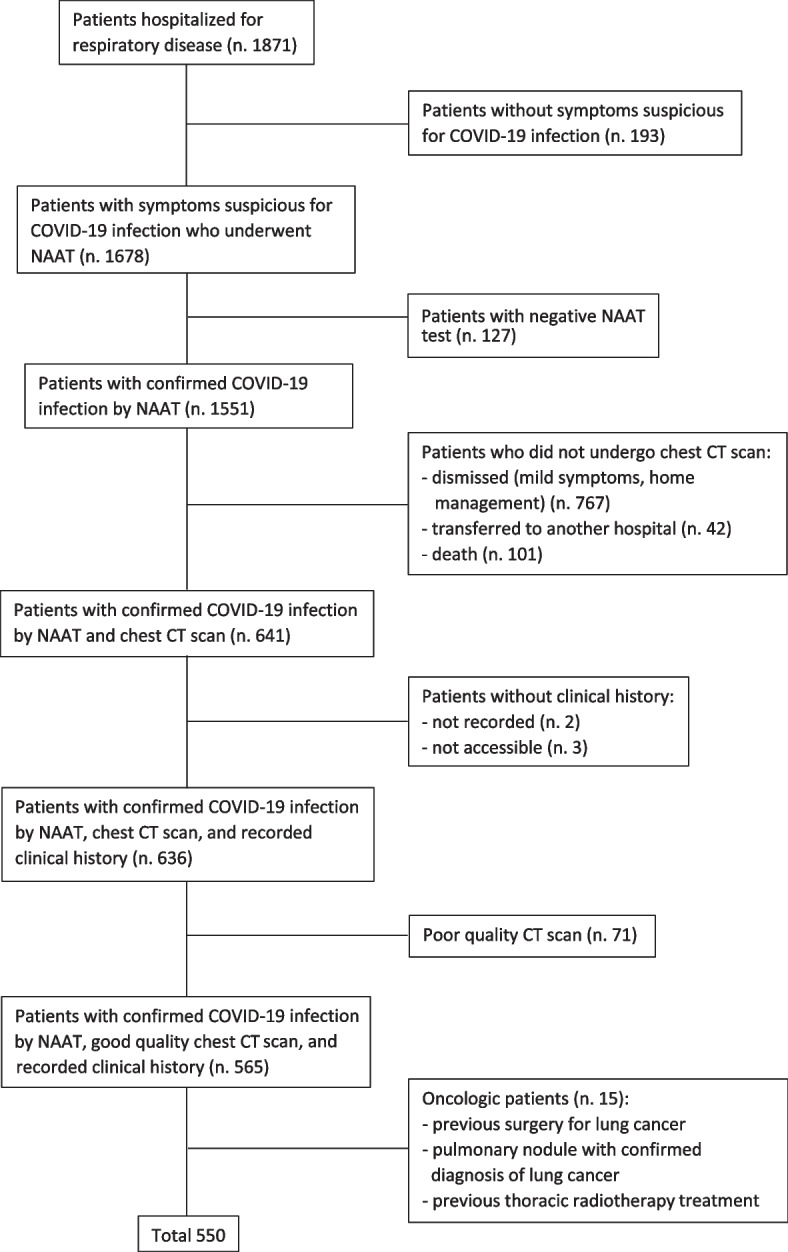
Flow-chart for patients’ selection

In this cohort study, clinical history and imaging findings of all patients aged ≥18 years diagnosed with COVID-19, admitted to Careggi University Hospital (Florence) from 1 March 2021 and 30 April 2022 were retrospectively reviewed. From the originally enrolled sample made up of 1871 patients (Fig. 1), several were excluded according to the following criteria:

- No confirmed COVID-19 infection by NAAT.
- No chest CT examination during hospitalization.
- Poor CT imaging quality.
- No clinical history before and during hospitalization.
- Previous thoracic radiotherapy treatment.
- Previous surgery for lung cancer.
- Oncologic patient.

The final sample consisted of 550 patients (range 25–98 years; 354 males, mean age 66.1; 196 females, mean age 70.9). Study population was furtherly subdivided into three groups: 25–64 years (adults), 65–79 years (older adults), > = 80 years (oldest-old adults) [16]. The study was conducted according to the guidelines of the Declaration of Helsinki. This was a monocentric retrospective comparative study approved by the local Ethical Review Board (#18085/OSS). All patients gave their informed consent to undergo CT examinations and participate to this research protocol.

### Imaging

Chest CT examinations were carried out with a 128-detector row helical CT scanner (Somatom Definition AS, Siemens Healthcare, Erlangen, Germany) with the following parameters: tube voltage 100 kV, pixel size 0.465 mm, both slice thickness and reconstruction 1 mm, current *×* exposure time 150 mAs, rotation time 0.3 s, pitch 1.2 mm, and reconstruction kernel Bf70 (very sharp). Scans were performed in full inspiration with patient in supine position. Field of view was extended from lung apexes to bases. Post-processing, 1-mm-thick sections were obtained on axial, sagittal, and coronal planes oriented on the tracheal plane. Intravenous contrast agent was not administered. Chest CT images were displayed on a 22-in. medical monitor with a 3-megapixel Barco display (Barco, Kortrijk, Belgium) and 2048 × 1536 resolution. The software programs originally provided with the systems were used for image assessment.

### Study design, CT scores and features

To summarize the many doubts and uncertainties arising from clinicians and radiologists during MDT, ten FAQ initially developed were condensed into four points (Additional file 1*, section a*). The selection was made on the basis of the three following reasons:

- frequency with which questions arose during MDT;
- possibility of this research to answer questions with confidence and clarity;
- implications that those answers could have had on the therapeutic strategy.

The issues listed are not intended to be hierarchical or all-inclusive but cover the major issues related to COVID19, imaging data, and outcomes. This research specifically investigated the relationship between:

1. CO-RADS, chest CT score, clinical score, and outcome;
2. the involvement of a specific lung lobe and outcome;
3. dominant pattern/distribution and severity score, for the same chest CT score;
4. additional factors and outcome.

In order to answer such questions, this study included the first chest CT examination carried out by each patient during the hospitalization independently of the time elapsed between the onset of symptoms or positive NAAT result and chest CT examination to allow the correlation of CT scores with different stages and disease progression. This way, patients with severe clinical conditions were also included allowing to investigate CT abnormalities linked with disease severity. CT images were assessed for scan quality considering motion artifacts and inspiratory level. The 7 parameters we adopted to investigate and assess each CT examination, are below reported [11, 17, 18]:

- **A) -** CO-RADS scores based on COVID-19 lung involvement ranged from 1 to 5, with higher values reflecting an increase in likelihood of patients having confirmed COVID-19 with lung involvement, whereas lower values were attributed to CT examinations either normal or showing abnormalities less likely linked to COVID-19 infection in which an alternative diagnosis was more probable [19]. CO-RADS is a useful tool used to diagnose COVID-19, especially when NAATs are not readily available [6]. The 5-score CO-RADS scale (Table 1) is summarized as follows [6]: 1: very low level of suspicion; 2: low level of suspicion; 3: equivocal findings; 4: high level of suspicion; 5: very high level of suspicion.
- **B) -** Chest CT score for single lobe involvement. 0: 0%; 1: < 5%; 2: 5–25%; 3: 26–50%; 4: 51–75%; 5: > 75%.
- **C) -** Dominant pattern (consolidation, ground-glass opacity, multifocal, crazy-paving, or reverse halo), defined by the Fleischner Society (Additional file 1, section b) [20].
- **D) -** Dominant distribution (lower lobes, peripheral, bronchocentric, dorsal, apical, or diffuse).
- **E) -** Additional COVID-19 related findings (vascular enlargement, pleural thickening, air bubble sign, halo sign, subpleural sign, perilobular pattern, and subpleural sparing) [21–25].
- **F) -** Additional findings not typical for COVID-19 (pleural effusion, pericardial effusion, discrete small nodules, tree-in-bud, cavitation, isolated lobar/segmental consolidation, smooth interlobular septal thickening, atelectasis, and lymphadenopathy) [13, 14, 26].
- **G) -** Comorbidities at CT (cardiomegaly, aortic/coronaric calcifications, pre-existing pulmonary disease, steatosis, and tracheomalacia).

**Table 1:** CO-RADS score scale

CO-RADS score	Diagnostic suspicion	Findings
CO-RADS 1	Normal or non-infectious etiology	No abnormalities or emphysema, perifissural nodules, lung tumors, fibrosis
CO-RADS 2	Infectious etiology not compatible with COVID-19	Tree-in-bud, centrilobular nodular pattern, lobar or segmental consolidation, lung cavitation
CO-RADS 3	Equivocal findings	Perihilar ground-glass, homogenous extensive ground-glass with or without sparing of some secondary lobules, or ground-glass with interlobular septal thickening in absence of other typical CT finding
CO-RADS 4	Typical for COVID-19 but with some overlap with other viral pneumonias	Similar to CO-RADS 5 but not located in contact with visceral pleura or are located strictly unilaterally, are in a predominant peribronchovascular distribution or superimposed on severe pre-existing pulmonary abnormalities
CO-RADS 5	Typical for COVID-19	*Mandatory features:* ground-glass opacities, with or without consolidations, close to visceral pleura, including fissures, and multifocal bilateral distribution *Confirmatory pattern (at least one):* crazy-paving, consolidations, organizing pneumonia

### Clinical score and outcomes

The following clinical data were collected and recorded anonymously: age, gender, date of admission, disease severity at the date of chest CT examination, admission to and length of stay in the ICU, and date of death if present. The World Health Organization Clinical Progression Scale was used to assess the disease severity. It is a scoring scale developed as a common tool to be used in clinical studies of COVID-19 that evaluates the clinical progression of the infection based on treatment intensity [27]. The scale ranges from 0 (virus-free) to 10 (dead) with increasing numbers reflecting a worsening of clinical conditions, often meaning an increase in the need of ventilatory support (Additional file 1*, section c*).

For statistical purposes, we simplified such classification grouping patients with similar scores – exactly the first 4 scores and the last 3 scores into two categories – getting a classification subdivided into 6 categories (Table 2).

**Table 2:** Clinical score and categories proposed in the current study

Clinical score	Definition	Category
0	uninfected	**1**
1	asymptomatic	
2	symptomatic, independent	
3	symptomatic, assistance needed	
4	hospitalized, no oxygen therapy	**2**
5	hospitalized, oxygen by mask or nasal prongs	**3**
6	hospitalized, oxygen by non-invasive ventilation or high-flow	**4**
7	intubation and mechanical ventilation, pO_2_/FiO_2_ ≥ 150 or SpO_2_/FiO_2_ ≥ 200	**5**
8	mechanical ventilation pO_2_/FiO_2_ < 150 (SpO_2_/FiO_2_ < 200) or vasopressors	**6**
9	mechanical ventilation pO_2_/FiO_2_ < 150 and vasopressors, dialysis or ExtraCorporeal Membrane Oxygenation	
10	dead	

Disease outcome was defined as one of the following three conditions: 1) Discharge after hospital admission in ordinary ward; 2) Discharge after admission in ICU; 3) Death.

Patients’ follow-up lasted until their discharge (after admission to ICU or not) or death.

### Readers and statistical analysis

All chest CT examinations were assessed by two ten-year-experienced in chest imaging radiologists (EC-VR). Whenever they disagreed, a decision was made in consensus after discussion. Cohen’s Kappa statistics was used to estimate the agreement between the two readers in classifying the patients into the categorical variables defined by CO-RADS score, chest CT score for lobe involvement, dominant pattern, dominant distribution, additional findings, and comorbidities at CT. Cohen’s K-values were considered as a measure of inter-reader agreement as follows: 0.01–0.20 weak; 0.21–0.40 poor, 0.41–0.60 moderate, 0.61–0.80 good, 0.81–0.99 excellent, and 1 perfect.

Data was presented as percentages/mean (± standard deviation) and median (interquartile range). The Kolmogorov-Smirnov test was used as a goodness-of-fit test for normality. To answer the research questions, different statistical analyses were performed depending on normality and data type: association between categorical data was tested using Chi^2^ test; correlation between numerical data was evaluated using Pearson or Spearman correlation analysis (depending on normality); association between numerical and categorical data was assessed using ANOVA or Kruskal-Wallis test (depending on normality). For all the analyses, a *p*-value < 0.05 was considered as statistically significant. The collected data were analyzed using the IBM SPSS® v. 28.0 statistical analysis software (IBM Corp., New York, NY, USA).

## Results

The inter-reader agreement for the categorical variables was good or excellent depending on the parameter investigated. In particular, the inter-reader agreement was excellent for CO-RADS (K = 0.83), dominant pattern (K = 0.88), additional findings (K = 0.94), and collateral findings (K = 0.96), whereas it was good for chest CT score for lobe involvement (K = 0.74) and dominant distribution (K = 0.76).

Of the 550 patients included in this study, 311 were discharged after stay in ordinary ward, 151 after admission to ICU and 88 died during hospitalization. Median and mean ICU length of stay were 16 and 22 days respectively. Median and mean time between hospitalization and death were 24 and 26 days respectively.

Population data are summarized in Table 3. Further data are available in the additional file (Additional file 1, *section d).*

**Table 3:** Population data

Variables		N	%
Sex	Males	196	35.6
	Females	354	64.4
Age group	25–64	226	41.1
	65–79	282	33.1
	> = 80	142	25.8
Outcomes	Ordinary hospitalization	310	56.4
	ICU and alive	151	27.5
	Death	89	16.2

*ICU* intensive care unit

Significant association exists between age and outcome: the older the patients the worse the outcome (Table 4).

**Table 4:** Association between patients age for group and outcome

			Outcome			Total
			Ordinary hospitalization	ICU and alive	Death	
Age group	25–64	N	139 (61.5%)	68 (30.1%)	19 (8.4%)	226 (100%)
	65–79	N	102 (56.0%)	48 (26.4%)	32 (17.6%)	182 (100%)
	> = 80	N	69 (48.6%))	35 (24.6%)	38 (26.8%)	142 (100%)
Total		N	310 (56.4%)	151 (27.5%)	89 (16.2%)	550 (100%)

Chi2 test, *p* < 0.001

Comorbidities detected at CT were significantly associated with outcome, in particular, death and ICU admission were more frequent among patients with cardiomegaly, coronaric calcification or steatosis. On the contrary, outcome is less severe when no comorbidities have been detected. Pre-existing pulmonary diseases or tracheomalacia did not significantly affect the outcome (Table 5).

**Table 5:** Association between comorbidities detected at CT scan and outcome

Comorbidities at CT	Outcome			Total	P (Chi2 test)
	Ordinary hospitalization	ICU and alive	Death		
None	71 (59.7%)	41 (34.5%)	7 (5.9%)	119 (100%)	0.002
Cardiomegaly	25 (45.5%)	12 (21.8%)	18 (32.7%)	55 (100%)	0.002
Coronaric calcification	91 (45.7%)	59 (29.6%)	49 (24.6%)	199 (100%)	< 0.001
Pre-existing pulmonary disease	17 (47.2%)	10 (27.8%)	9 (25.0%)	36 (100%)	0.279
Steatosis	22 (31.9%)	34 (49.3%)	13 (18.8%)	69 (100%)	< 0.001
Tracheomalacia	5 (35.7%)	6 (42.9%)	3 (21.4%)	14 (100%)	0.307
Total	310 (56.4%)	151 (27.5%)	89 (16.2%)	550 (100%)

1. **Which was the relationship between CO-RADS, chest CT score, clinical score, and outcomes?**

A strong significant correlation emerged between CO-RADS and chest CT score, without differences between lobes (rho values ranged from 0.505 to 0.584). CO-RADS also showed a significant positive correlation (rho> 0.3) with clinical score and outcome parameters, but not with the length of ICU stay (*p* > 0.05). Additionally, chest CT score had strong correlation (rho > 0.5) with clinical score and outcome parameters. In particular, when chest CT score was > 50 and > 75%, death occurred in around one over four and one over three patients, respectively. In synthesis, when chest CT findings were suggestive for COVID-19 infection in NAAT positive patients, lobe involvement correlated with clinical score, and prognosis without differences between lobes (Table 6).

**Table 6:** Spearman correlation analysis between CO-RADS, chest CT score, clinical score, outcome, and intensive care unit duration

CO-RADS and Chest CT score	Statistical parameter	Clinical Score (at CT scan date)	Outcome	ICU Stay Duration
CO-RADS	Rho	0.334	0.18	0.097
	P	< 0.001	< 0.001	0.152
	N	550	549	219
Chest CT score Left Upper Lobe	Rho	0.518	0.320	0.260
	P	< 0.001	< 0.001	< 0.001
	N	550	549	219
Chest CT score Left Lower Lobe	Rho	0.548	0.346	0.315
	P	< 0.001	< 0.001	< 0.001
	N	550	549	219
Chest CT score Right Upper Lobe	Rho	0.538	0.344	0.335
	P	< 0.001	< 0.001	< 0.001
	N	550	549	219
Chest CT score Right Middle Lobe	Rho	0.532	0.348	0.290
	P	< 0.001	< 0.001	< 0.001
	N	550	549	219
Chest CT score Right Lower Lobe	Rho	0.558	0.343	0.312
	P	< 0.001	< 0.001	< 0.001
	N	548	547	217

*ICU* intensive care unit

2) **Was there any association between the involvement of a specific lobe and outcomes?**

For each lobe, significant associations were found between the percentage of involvement and the outcome – the higher the involvement, the higher the percentage of patients with worse outcomes – but no significant differences were found among lobes. Indeed, no significant association was found between the involvement of a specific lobe with any outcome measure, except the one between high percentage of right middle lobe involvement (50 and 75%) and a relevant death rate (Chi^2^ test, *p* < 0.005). Specifically, in case of right middle lobe involvement lower than 50%, death rate was less than 10%, whereas it increased to 30 and 37% for lobar involvement greater than 50 and 75% respectively. Moreover, right middle lobe involvement greater than 50% was correlated with higher median length of ICU stay (Table 7).

**Table 7:** Association between Chest CT Score (> 50 and > 75%) and outcome and Intensive Care Unit stay duration

Chest-Severity Score	Ordinary hospitalization*			ICU admittance*		Death*		Total	Days in ICU°		Total
	N	%		N	%	N	%	N	mean ± SD	median [IQR]	N
> 50%	LUL	74	35.4	81	38.8	53	25.4	208	24.4 ± 18.7	19 [10; 33]	127
	LLL	98	38.4	96	37.6	60	23.5	254	24.7 ± 18	20 [10; 33]	147
	RUL	82	37.1	79	35.7	59	26.7	220	25.3 ± 18.8	20 [10.5; 35.5]	129
	RML	65	34.6	65	34.6	57	30.3	187	25.3 ± 18	22 [11; 33.2]	114
	RLL	111	41.1	97	35.9	61	22.6	269	24.7 ± 18	20 [10.5; 33]	149
> 75%	LUL	23	24.2	43	45.3	29	30.5	95	26.1 ± 19.6	20 [11; 34]	69
	LLL	44	30.6	59	41.0	41	28.5	144	25.9 ± 18.7	22 [11.7; 33.5]	98
	RUL	28	25.9	42	38.9	38	35.2	108	29 ± 19.8	26 [14; 40]	75
	RML	22	23.4	37	39.4	35	37.2	94	25.5 ± 17.6	23 [12; 32.5]	69
	RLL	43	30.3	55	38.7	44	31.0	142	26.4 ± 19	22 [12; 36]	95

*LUL* left upper lobe, *LLL* left lower lobe, *RUL* right upper lobe, *RML* right middle lobe, *RLL* right lower lobe, *SD* standard deviation, *IQR* interquartile range, *ICU* intensive care unit

*Chi2 test, *p* < 0.001; °Kruskal-Wallis test, *p* < 0.001

To investigate for the existence of a bias, it was evaluated whether right medium lobe involvement was strongly associated with the involvement of other lobes. As expected, right medium lobe involvement was related to involvement of all other lung lobes, especially right lower lobe (Chi^2^ test, p < 0.05). When right middle lobe involvement was greater than 75%, right lower lobe involvement was greater than 75% too (87% of cases, to be exact). Conversely, right lower lobe involvement greater than 75% was associated to severe right middle lobe involvement in just over half of cases (Table 8).

**Table 8:** Association between right middle lobe and other lobes

Chest CT score	Left upper lobe		Left lower lobe		Right upper lobe		Right lower lobe	
	N	%	N	%	N	%	N	%
5–25%	3	3.2	2	2.1	1	1.1	0	0
25–50%	7	7.4	3	3.2	3	3.2	0	0
50–75%	22	23.2	4	4.2	15	15.8	0	0
> 75%	63	66.3	86	90.5	76	80.0	95	100
Total	95	100	95	100	95	100	95	100

Involvement of the left upper, left lower, right upper, and right lower lobes among patients with right middle lobe involvement greater than 75% (*N* = 95). For each lobe, *p* < 0.05 (Chi^2^ test)

3) **Was the dominant pattern or distribution relevant for the same chest CT score?**

When lobe involvement was lower than 50%, dominant pattern or distribution was not relevant. For all lung lobes except the right upper lobe, crazy-paving as dominant pattern was related to high death rate when lobar involvement was greater than 50% (Table 6). In particular, 15% more patients died when crazy-paving was the dominant pattern almost doubling the death rate with respect to patients without such pattern: 35.9% vs 19.2% for left upper lobe involvement > 50%, 31.5% vs 19.4% for left lower lobe, 41.4% vs 23.9% for right middle lobe, and 32.2% vs 17.9% for right lower lobe (Table 9).

**Table 9:** Association between Chest CT score, “crazy paving” pattern, and outcome for lobe involvement greater than 50%

Chest CT score	Crazy paving	Ordinary N (%)	Intensive Care Unit and alive N (%)	Dead N (%)	Total N (%)
LUL > 50%	No	53 (40.8%)	52 (40.0%)	25 (19.2%)	130 (100%)
	Yes	21 (26.9%)	29 (37.2%)	28 (35.9%)	78 (100%)
LLL > 50%	No	72 (43.6%)	61 (37.0%)	32 (19.4%)	165 (100%)
	Yes	26 (29.2%)	35 (39.3%)	28 (31.5%)	89 (100%)
RML > 50%	No	48 (41.0%)	41 (35.0%)	28 (23.9%)	117 (100%)
	Yes	17 (24.3%)	24 (34.3%)	29 (41.4%)	70 (100%)
RLL > 50%	No	85 (47.5%)	62 (34.6%)	32 (17.9%)	179 (100%)
	Yes	26 (28.9%)	35 (38.9%)	29 (32.2%)	90 (100%)

Right upper lobe is not shown since no specific pattern showed significant correlation with outcome. LUL: left upper lobe. LLL: left lower lobe. RML: right middle lobe. RLL: right lower lobe. For each lobe, *p* < 0.05 (Chi^2^ test)

4) **Did the “additional factors” typical or contraindicative of COVID-19 matter?**

Few positive significant correlations between additional factors and outcome emerged. In addition, Pearson correlation analysis revealed weak relationships between the variables, as indicated by low rho values. Nonetheless, some significant associations were found (p < 0.001), as follows:

- the presence of pleural effusion with rates of ICU admission (41.4% vs 24.0%) or patient death (17.1% vs 15.8%), nevertheless with no clinical score change (Table 10);
- the presence of lymphadenopathies with outcome measures, especially high rate of ICU admission (33.3% vs 25.5%) or patient death (24.6% vs 13.1%) (Table 10).

**Table 10:** Association between additional findings and outcome

Additional findings contraindicative for COVID19		Outcome			Total N (%)
		Ordinary N (%)	ICU and alive N (%)	Dead N (%)	
Pleural effusion*	No	264 (60.3%)	105 (24.0%)	69 (15.8%)	438 (100%)
	Yes	46 (41.4%)	46 (41.4%)	19 (17.1%)	111 (100%)
Lymphadenopathy*	No	252 (61.3%)	105 (25.5%)	54 (13.1%)	411 (100%)
	Yes	58 (42.0%)	46 (33.3%)	34 (24.6%)	138 (100%)
Total		310 (56.5%)	151 (27.5%)	88 (16.0%)	549 (100%)

*ICU* intensive care unit. * Chi^2^ test, *p* < 0.001

## Discussion

Chest CT score correlated both with clinical score and prognosis, thus enabling to attempt an answer to the main FAQ arising from our hospital meetings on COVID-19.

This series, to our best knowledge, is one of the largest with detailed clinical/radiological data and allowed the investigation of correlations or associations previously not explored. CT scans were not performed at the same time after symptoms onset. Nowadays there is not an exact cut-off that allows to differentiate CT examinations in the early stage from advanced stage of the disease, since CT abnormalities increase gradually over the first 2 weeks after the initial onset of symptoms [28]. Peak levels of lung involvement are reached 6–11 days from symptom onset, and peak severity of respiratory manifestations occurs approximately 10–15 days after symptom onset. Usually, possible improvements can be detected on CT scans after 14 days [29]. Therefore, patients should undergo chest CT examinations after 5 days of symptoms onset to allow the detection of acute parenchymal changes at their peak [30]. It could be hypothesized that only those CT scans obtained after 5 days of symptoms onset, had to be included in our study. However, this study included chest CT scans performed at different times from symptom onset allowing the evaluation of parenchymal changes at different stages of the disease [31].

**Fig. 2:**
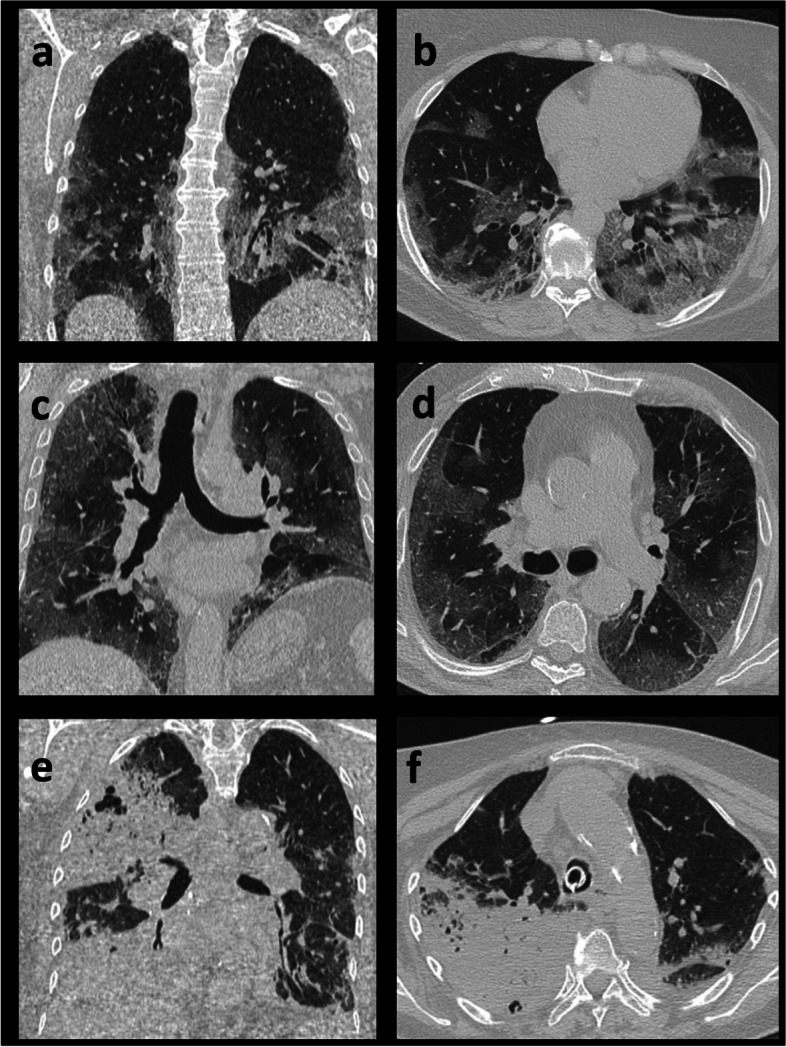
Chest CT scans in axial and coronal planes for: **a**, **b** crazy-paving pattern; **c**, **d** extensive lung involvement with predominantly ground-glass opacities; **e**, **f** consolidation with air bronchogram of the right middle lobe extending to the right superior lobe

In the first point, dealing about the relationship among clinical, imaging scores and outcome, the current study confirmed on a wider numerical sample what was stated by Li K. et al. and Francone M. et al. [10, 11] on the correlation between chest CT score and disease severity. In addition, it was demonstrated that chest CT score yields a prognostic value too (rho > 0.5). When chest CT score was greater than 50 and 75%, death occurred in around one over four and one over three patients, respectively (Fig. 2c-d). Furthermore, CO-RADS showed a good correlation with clinical score and prognosis (rho> 0.3). CO-RADS was developed to determine the probability and not the severity of COVID-19. However, probably due to the virus aggressiveness, the greater was the probability that lung involvement was COVID-19 related, the worse was outcome.

The second question – the association between the involvement of a specific lobe and outcome – lead, for the first time since COVID-19 outbreak, to analyze lobe independently for a relationship between involvement and disease severity. Interestingly, it emerged that middle lobe involvement (Fig. 2e-f) is a very unfavorable prognostic criterion and is related to greater disease severity. When its rate of involvement is less or more than 50%, 1 patient or 3 patients out of 10 will face death, respectively. This is somewhat unexpected since middle lobe is the smallest lobe and should contribute less to lung function. Indeed, it emerged that middle lobe is frequently associated with an extensive lung involvement. Specifically, in almost 9 out of 10 cases, severe right middle lobe involvement (> 75%) was associated with severe right lower lobe involvement (> 75%). The use of classifications as the chest CT score, considering single lobe involvement rates should be considered too laborious and time consuming. A visual assessment of the percentage of the total lung volume involvement might be sufficient. Alternatively, a software for quantitative analysis of chest CT abnormalities, such as a deep learning software called LungQuant could be implemented to speed up its evaluation [32].

Regarding the third question – whether the dominant pattern/distribution for the same chest CT score is significant or not with respect to the outcome – our data showed that when disease burden is less than 50%, the presenting pattern does not influence prognosis or clinical severity, but, when lung involvement is extensive, crazy-paving (Fig. 2a-b) leads to a worse prognosis and about 15% more patients will die with respect to other patterns. In other words, if lung involvement is more than 50% and the dominant pattern is not crazy paving, we can expect a 15% less death rate. This finding agrees with that by Li K et al., Elmokadem et al. and Meiler at al [10, 33, 34]. They observed a correlation between such pattern and disease severity. However, at variance with Li K et al. [10], no association was found between the presence of consolidation and illness severity, reflecting that it is not necessary to achieve a complete alveolar filling to significantly impair lung function.

Finally, it is known that COVID-19 determines vessel inflammation and thrombosis, which cannot be detected on non-contrast enhanced CT scan. As noted by Li K et al. [10], the presence of ground-glass opacities did not show any correlation with illness severity. This is explainable because ground-glass opacities can be caused either by mild-alveolar edema, interstitial thickening, protein exudate, or severe-alveolar diffuse damage with cellular fibromyxoid exudate.

Lastly, the fourth point, concerning additional factors, revealed that they were, in this series, irrelevant or nearly. It emerged that almost none of them clinical severity or prognosis. Only the presence of lymphadenopathies and pleural effusion can worsen prognosis since the rate of ICU admission increases from 24.0 to 41.4% and 13.1 to 24.6%, respectively. Lymph-nodes enlargement is probably a marker of a greater degree of inflammation, whilst pleural effusion, which is atypical in COVID-19, determines a reduction of lung volume and thus impairs patients’ ventilation. Overall, these findings are consistent with those observed by Li Kunwei et al. [10].

### Limitations

This study has some limitations, the main being that no further clinical or radiological data of admitted patients was obtained after discharge. Therefore, without a longitudinal observational study, the long-term impact of CT findings on patients remains undetermined.

Another limitation was the lack of comparative CT examinations performed before the infection excluding the absence of pre-existing small airway disease and lung parenchymal fibrotic-like changes at the root of architectural distortions such as consolidations, honeycombing, traction bronchiectasis, and volume loss. Additionally, the present study involved patients in a relatively short period of time of the COVID-19 pandemic and thus it was not possible to make a comparison between different waves of the disease. Since the virus has been evolving over time with the breakout of several variants associated to different contagiousness and severity in disease extension, further studies are needed to investigate the impact of different variants on chest CT findings and outcome. Pharmacological treatments did not constitute an interfering bias since patients enrolled in 2021–2022 were treated with drugs that had already demonstrated efficacy against COVID-19 infection and its complications. This observation strengthened the correlations between CT features and patients’ outcome without creating interferences due to poorly effective or “pioneering” treatments.

Vaccination and immunization status were not known; therefore, it remains undetermined if and how previous exposure to the virus or vaccination could have influenced the imaging presentation of the disease. Finally, no sure prediction of COVID-19 patients’ course can be made exclusively based on radiological data emerged from the current study.

## Conclusions

In our series: 1) If CT was suggestive for COVID (NAAT+), there was a strong correlation between CT/clinical score and prognosis; 2) Middle lobe CT involvement was an unfavorable prognostic criterion; 3) If CT score was lower than 50%, the pattern was not influential, whereas if CT score was greater than 50%, “crazy paving” as dominant pattern led to a 15% increased death rate, thus almost doubling it; 4) Additional factors did not matter, but lymph-nodes enlargement and/or pleural effusion worsen prognosis.

### Supplementary Information

**Additional file 1.** Supplemental material.

## Data Availability

The datasets analyzed during the current study are available from the corresponding author on reasonable request.

## Abbreviations

CO-RADS: COVID-19 Reporting and Data System
ECMO: Extra Corporeal Membrane Oxygenation
FAQ: Frequently Asked Questions
ICU: Intensive Care Unit
NAAT: Nucleic Acid Amplification Test
NIV: Non Invasive Ventilation
