# Supplementary material for: Doubts and concerns about COVID-19 uncertainties on imaging data, clinical score, and outcomes
Source: BMC Pulm Med. 2023 Nov 25;23:472. doi: 10.1186/s12890-023-02763-3 (PMC10675953; doi:10.1186/s12890-023-02763-3)
Supplement: Supplementary file 1 — Additional file 1. Supplemental material. [file 12890_2023_2763_MOESM1_ESM.docx]

**Supplemental material**

***Section a***

Ten initially selected questions on COVID-19:

1. What is the relationship between CO-RADS and chest CT score?
2. What is the relationship between CO-RADS and outcome?
3. What is the prevalent chest CT score in COVID-19 patients?
4. What is the relationship between chest CT score and outcome?
5. Is there any correlation between the involvement of a specific lobe and outcome?
6. What is the role of chest CT score?
7. Is the dominant pattern relevant for the same chest CT score?
8. Is the dominant distribution relevant for the same chest CT score?
9. What is the relationship between clinical score and outcome? (Is clinical score valid?)
10. How are the “additional factors” distributed?

Then, six questions on COVID-19:

1. What is the relationship between CO-RADS and chest CT score?
2. What is the relationship between CO-RADS and clinical score and outcome?
3. What is the relationship between chest CT score and clinical score and outcome?
4. Is there any correlation between the involvement of a specific lobe and outcome?
5. Is the dominant pattern or distribution significant for the same chest CT score?
6. How are the “additional factors” distributed?

Finally, four condensed questions on COVID-19:

1. Which is the relationship between CO-RADS, chest CT score, clinical score, and outcomes?
2. Is there any association between the involvement of a specific lobe and outcomes?
3. Is the dominant pattern or distribution relevant for the same chest CT score?
4. Do the “additional factors” typical or contraindicative of COVID-19 matter?

***Section b***

*Consolidation*: homogeneous increase in pulmonary parenchymal attenuation that obscures the margins of vessels and airway walls.
*Ground-glass opacity*: hazy increased opacity of lung, with preservation of bronchial and vascular margins.
*Crazy-paving pattern*: thickened interlobular septa and intralobular lines superimposed on a background of ground-glass opacity, resembling irregularly shaped paving stones.
*Reversed halo sign*: focal rounded area of ground-glass opacity surrounded by a more or less complete ring of consolidation.

***Section c***

Patients’ medical records were examined to assess the treatment intensity the same day of the CT examination and a score was assigned as follows. 0: uninfected; 1: asymptomatic; 2: symptomatic, independent; 3: symptomatic, assistance needed; 4: hospitalized, no oxygen therapy; 5: hospitalized, oxygen by mask or nasal prongs; 6: hospitalized, oxygen by Non Invasive Ventilation or high-flow; 7: intubation and mechanical ventilation, pO_2_/FiO_2_ ≥150 or SpO_2_/FiO_2_ ≥200; 8: mechanical ventilation pO_2_/FiO_2_ <150 (SpO_2_/FiO_2_ <200) or vasopressors; 9: mechanical ventilation pO_2_/FiO_2_ <150 and vasopressors, dialysis or ECMO; 10: dead.

***Section d***

**Additional Table 1.**

| Chest CT score | Left Upper Lobe | Left Lower Lobe | Right Upper Lobe | Right Middle Lobe | Right Lower Lobe | Total |
| --- | --- | --- | --- | --- | --- | --- |
| 0% | 35 (6,4%) | 30 (5,5%) | 39 (7,1%) | 40 (7,3%) | 27 (4,9%) | 171 (10,6%) |
| 0-5% | 59 (10,7%) | 34 (6,2%) | 52 (9,5%) | 70 (12,7%) | 35 (6,2%) | 250 (9,1%) |
| 5-25% | 147 (26,7%) | 105 (19,1%) | 124 (22,5%) | 131 (23,8%) | 87 (15,8%) | 594 (21,6%) |
| 25-50% | 100 (18,2%) | 126 (22,9%) | 114 (20,7%) | 121 (22,0%) | 130 (23,6%) | 591 (21,5%) |
| 50-75% | 114 (20,7%) | 110 (20%) | 112 (20,4%) | 93 (16,9%) | 128 (23,1%) | 557 (20,3%) |
| >75% | 95 (17,3%) | 145 (26,4%) | 109 (19,8%) | 95 (17,3%) | 143 (26,0%) | 587 (21,3%) |
| Total | 550 (100%) | 550 (100%) | 550 (100%) | 550 (100%) | 550 (100%) | 2750 (100%) |

**Additional table 1.** Summary of frequencies of chest CT score for each lobe. Percentages are shown in brackets.
